# Bioactive Polymer Materials with Antibacterial Properties: An Editorial

**DOI:** 10.3390/polym17030394

**Published:** 2025-02-01

**Authors:** Md. Amdadul Huq, Shahina Akter

**Affiliations:** 1 Department of Food and Nutrition, College of Biotechnology and Natural Resource, Chung-Ang University, Anseong-si 17546, Republic of Korea; 2 Department of Food Science and Biotechnology, Gachon University, Seongnam 13120, Republic of Korea; shahinabristy16@gmail.com

Bioactive materials have a wide range of applications, and bioactive materials with antibacterial properties, in particular, have attracted significant medical interest [1]. Antibacterial bioactive materials can be obtained from nature or can be synthesized in different ways. They can be prepared via the simple combination of antibacterial substances with materials such as metals or polymers and applied through delivery strategies to kill pathogenic bacteria. The emergence of multidrug-resistant (MDR) bacteria due to the uncontrolled, immoderate, and multiple use of antibiotics and chemotherapeutics is a serious threat to the world population [2], as antibiotic-resistant microorganisms cause life-threatening diseases in humans. The development of novel, safe, and effective antibacterial agents is the decisive solution to this issue. In this context, bioactive polymer materials with antibacterial properties could be promising agents to control MDR bacteria [3]; therefore, this Special Issue focuses on such materials. It is an open forum where scientists/researchers may share their knowledge, investigations, and findings in this promising field.

This Special Issue contains a total of 17 articles. Among these 17 articles, 3 are comprehensive review articles, and the remaining 14 are research articles. One review article in this Special Issue provides an overview of the green synthesis of AgNPs using different biological resources; the various parameters essential for stable, easy synthesis and high yields; the antibacterial applications and mechanisms of biosynthesized AgNPs; and the prospects for their future development and potential antibacterial applications [4]. Another review article in this Special Issue describes the biosynthesis of chitosan-coated polymeric silver and gold nanoparticles, their characterization, and their potential antibacterial applications. The authors also highlight various mechanisms of the biosynthesized chitosan-coated polymeric silver and gold nanoparticles against pathogenic bacteria [3]. The third review article in this Special Issue focuses on recent advances in natural polymer-based hydrogels for antibacterial wound therapeutics [5].

Fourteen original articles on this research topic are devoted to the antimicrobial applications and mechanisms of various bioactive materials against pathogenic microorganisms. Wang et al. [6] describe the probiotic-mediated biosynthesis of silver nanoparticles (AgNPs) and discover their potent antimicrobial activity against pathogenic strains of *Escherichia coli* O157:H7. Their study suggests that the biosynthesized AgNPs could be used as an excellent new type of antimicrobial agent to control multidrug-resistant strains of *Escherichia coli* O157:H7 [6]. Umoren et al. [7] report the biosynthesis and characterization of chitosan–CuO nanocomposite and evaluate their antibacterial activity against various Gram-positive and Gram-negative bacteria. Their study demonstrates that the biogenic chitosan–CuO nanocomposite exhibits strong antimicrobial activity against the Gram-positive bacteria *Bacillus licheniformis*, *Bacillus cereus,* and *Micrococcus luteus* and the Gram-negative bacteria *Pseudomonas aeruginosa*, *Pseudomonas citronellolis*, *E. coli*, *Klebisiella* sp., *Bradyrhizobium japonicum*, and *Ralstonia pickettii*. Eom et al. [8] report that synthetic polypeptides with cationic arginine moieties show high antimicrobial activity against Gram-positive *B. subtilis* and Gram-negative *E. coli*. Wang and coworkers [9] conclude that the inclusion of curcumin in a biopolymer-based film transport system in combination with photodynamic activation represents a promising option for the preparation of food packaging films. Edis et al. [10] conduct research on antimicrobial biomaterials for sutures, bandages, and face masks with the potential for infection control. They conclude that the facile combination of *Aloe Vera Barbadensis* Miller (AV), trans-cinnamic acid (TCA), and iodine (I2) encapsulated in a polyvinylpyrrolidone (PVP) matrix seems a promising alternative to common antimicrobials. Nguyen and Vo [11] report the green synthesis of AgNPs using *Cordyline fruticosa* L. leaf extract as a reducing and capping agent and investigate their bactericidal effect against six pathogenic bacteria: *Escherichia coli*, *Pseudomonas aeruginosa*, *Salmonella enterica*, *Staphylococcus aureus*, *Bacillus cereus*, and *Enterococcus faecalis*. Their study demonstrates that the green synthesized AgNPs exhibit strong antimicrobial activity against the tested pathogenic bacteria. According to Latko-Durałek et al. [12], composites of polyamide 12 and metal oxides show high antimicrobial activity against *Escherichia coli*, *Candida albicans*, and *Herpes simplex* 1.

Nuanaon et al. [13] report the light-emitting-diode-assisted, fungal-pigment-mediated biosynthesis of silver nanoparticles, along with their antibacterial activity. Their study demonstrates that all LED-synthesized AgNPs exhibit antimicrobial potential against pathogenic *Escherichia coli* and *Staphylococcus aureus*. Prihandana et al. [14] investigate the antibacterial activity of a silver nanoflake (SNF)-blended polysulfone ultrafiltration membrane against *Escherichia coli* taken from river water. The SNFs show strong antimicrobial activity against the tested *Escherichia coli*. Roy et al. [15] evaluate the antimicrobial efficacy of quercetin against *Vibrio parahaemolyticus* biofilm on food surfaces and the downregulation of virulence genes. Their findings suggest that plant-derived quercetin should be used as an antimicrobial agent in the food industry to inhibit the establishment of *V. parahaemolyticus* biofilms. Schrekker and coworkers [16] describe the synthesis of high-density polyethylene–imidazolium salt (HDPE-IS) films and discover their potent antibiofilm activity against *Candida albicans, C. parapsilosis,* and *C. tropicalis*. Their study suggests that the synthesized HDPE-IS films demonstrate potential as biomaterials for the development of effective medical devices and tools that reduce the risk of fungal infections. Aguilar-Perez et al. [17] assess the effect of propolis on the antibacterial, mechanical, and adhesive properties of commercial poly(alkenoate) cement. They find that the modified cement shows high activity against *Streptococcus mutans*. Le et al. [18] develop antibacterial agents against *Escherichia coli* using the Maillard reaction product of chitosan and glucosamine prepared under gamma co-60 ray irradiation. Venkatesan et al. [19] report the synthesis and antimicrobial characterization of poly(butylene adipate-co-terephthalate)/kaolin clay biocomposites. Their study demonstrates that poly(butylene adipate-co-terephthalate)/kaolin clay biocomposites have great potential as food packaging materials due to their ability to decrease the growth of bacteria and improve the shelf life of packaged foods.

We hope that the reader will find useful references in this Special Issue for the development of novel, safe, and effective bioactive materials with antibacterial properties to control various drug-resistant pathogenic bacteria.

## Data Availability

Not applicable.
